# Electroacupuncture Reduces Body Weight by Regulating Fat Browning-Related Proteins of Adipose Tissue in HFD-Induced Obese Mice

**DOI:** 10.3389/fpsyt.2019.00353

**Published:** 2019-06-11

**Authors:** Sheng-Feng Lu, Yue-Xia Tang, Tao Zhang, Shu-Ping Fu, Hao Hong, Yu Cheng, Hou-Xi Xu, Xing-Yue Jing, Mei-Ling Yu, Bing-Mei Zhu

**Affiliations:** ^1^Key Laboratory of Acupuncture and Medicine Research of Ministry of Education, Nanjing University of Chinese Medicine, Nanjing, China; ^2^Huai’an Hospital of Traditional Chinese Medicine, Huaian, China; ^3^Regenerative Medicine Research Center, West China Hospital, Sichuan University, Chengdu, China

**Keywords:** electroacupuncture treatment, weight loss, adipose tissue browning, obese mice

## Abstract

**Objective:** This study investigated the influence of electroacupuncture (EA) and its potential underlying mechanisms on adipose tissue in obese mice.

**Methods:** Three-week-old male C56BL/6 mice were randomly divided to feed or not to feed high-fat diet (HFD), named HFD group and chow diet (CD) group, respectively. After 12 weeks, CD and HFD mice were randomly divided into two groups, respectively, to receive or not receive EA for 4 weeks. Body weight (BW) was monitored. Intraperitoneal glucose tolerance test and metabolic chamber recordings were performed. Blood samples and adipose tissue were collected for the analysis of leptin, triglyceride levels, and fat browning-related proteins.

**Results:** EA significantly reduced food intake, BW, and white adipose tissue (WAT)/BW ratio; decreased the adipocyte size and serum concentrations of triglyceride (TG) and cholesterol; and increased oxygen consumption in HFD mice. Compared with the CD mice, the HFD mice had elevated fasting serum glucose level and impaired glucose tolerance; however, these parameters were decreased by EA treatment. Meanwhile, EA promoted the protein and mRNA expressions of UCP1, PRDM16, and PGC-1α in adipose tissue, and activated sympathetic nerves *via* p-TH, A2AR, and β3AR in white adipose tissue.

**Conclusions:** EA reduced food intake, BW, TG, and cholesterol, and improved glucose tolerance in HFD mice. This ameliorative effect of EA on obesity-related symptoms associated with its promoted adipose tissue plasticity *via* activating sympathetic nerves.

## Introduction

Obesity and overweight are closely related to cardiovascular disease, type 2 diabetes mellitus, hypertension, and cancer, which have severely threatened human health and life and attracted much attention in many countries (1–3). Acupuncture for treatment of obesity in China for centuries is the most rapidly growing complementary and alternative therapy that is recognized by both the NIH and the WHO. In addition to reducing body weight (BW), body mass index, and waist-to-hip ratio effectively, there is a growing body of evidence showing that acupuncture improved obesity-associated dyslipidemia, leptin concentration, and inflammation, suggesting that acupuncture is as effective for obese individuals (4–7). Experimental studies also showed that acupuncture treatment effectively decreased BW of high-fat diet (HFD)-induced obese rats or mice by affecting the satiety center (8), the neuroendocrine system (4), and regulating inflammatory responses (9), although the underlying mechanism is not yet entirely clear.

Fat is the largest energy reserve in mammals. During periods of excessive caloric intake, almost excess energy is stored as triacylglycerol (TAG) in lipid droplets during lipogenesis. Under fasting conditions or high energy needs, the stored TAG in adipocytes is hydrolyzed into free fatty acids (FFAs) and glycerol *via* activation of lipolytic pathways (10). Catecholamines stimulate lipolysis through activating β-adrenergic receptors in target tissues, predominantly adipose tissue and muscle (11). Therefore, effectively activating sympathetic nervous system (SNS) and adipocyte metabolism has become an effective way to control obesity. In mammalian species, there are three types of adipocytes: white, beige/brite, and classical brown. They differ in lineage origin, morphology, abundance of mitochondria, number of lipid droplets, gene expression, and functions (12). In recent years, it has been observed that the adipose tissue is more dynamic than previously believed (13), especially the browning of white adipose tissue (WAT) in response to appropriate stimulation has aroused widespread interest and has become a new target for obesity therapeutics (14–17).

Our previous work implied that electroacupuncture (EA) can induce the expression of uncoupling protein-1 (UCP-1) in WAT (18) by stimulating Zusanli (ST36) and Neiting (ST44). Does EA stimulation induce WAT browning and adipose tissue plasticity? What is the possible mechanism involved? In this study, we employed HFD-induced obese mice as the animal model and treated them with EA on ST36 and ST44 acupoints. We aimed to observe the effect of EA on obesity and determine the expression of brown-related proteins, and then evaluate the level of adipose tissue plasticity and metabolic phenotype in EA-treated obese mice. Our results may provide evidence for the field to understand how EA clinically exerts its anti-obese role.

## Methods

### Animals and Grouping

Three-week-old male C57BL/6J mice (*n* = 63) were purchased from the Experimental Animal Center of Nanjing University of Chinese Medicine and were randomly divided into the common diet group (CD, *n* = 21) and the high fat diet food group (HFD, *n* = 42). Mice in the CD group were fed normal diet, and mice in the HFD group were fed D12451 Rodent Diet with 45 kcal% fat (supplied by Shanghai SLAC Laboratory Animal Co. Ltd). All mice were maintained at 24 ± 2°C in a 12-h light/dark cycle and given free access to water and food. Mice were weighted each week after 12 h of fasting. After 12 weeks, obese mice were defined by a 20% increase in total BW compared to control mice in the CD group and were then randomly divided into HFD (*n* = 9) and HFD + electro-acupuncture (EA) treatment group (HFD + EA, *n* = 14). Meanwhile, CD mice were randomly divided into the CD group (*n* = 8) and the CD + EA group (*n* = 13). This study was approved by the Institutional Animal Care and Use Committee of Nanjing University of Chinese Medicine and followed the latest NIH guidelines for the Care and Use of Laboratory Animals.

### EA Treatment

Mice in the CD + EA and HFD + EA groups were applied EA on ST36 and ST44 after physically restraining, while mice in the CD and HFD groups were restrained in the same way, without EA treatment. According to the standard published in Experimental Acupuncturology, ST36 is located in the anterior tibia muscle, about 3 mm distal to the knee joint, and ST44 is located between the second and third phalanges on the dorsum of the foot. For the EA mice, two stainless-steel needles 0.18 mm in diameter and 10 mm in length were separately inserted into each acupoint. An electric current was provided to the needles by a Han’s Acupoint Nerve Stimulator (Han Acuten, WQ1002F, Beijing, China), and EA frequency was set at 2/15 Hz with an intensity level of 1 mA for 30 min, once a day, 6 days per week, for a total of 4 weeks. The EA procedure was carried out with extremely gentle operation for avoiding any unnecessary stimulus and stress to the mice. BW and ingested food were monitored every week.

All of the mice were sacrificed with intravenous injection of high-dose pentobarbitone after 4 weeks of EA treatment, and samples were collected. The adipose tissue, including brown adipose tissue (BAT) in the interscapular region, epididymis WAT (Epi-WAT), and inguinal WAT (Ing-WAT), were dissected, weighted, and snap-frozen immediately in liquid nitrogen and stored at −80°C until further analysis.

### Morphological Analysis of White Adipose Tissue

The Epi-WAT tissues were fixed in 4% paraformaldehyde and embedded in paraffin, sectioned at 8-μm thickness. Hematoxylin and eosin staining (H&E staining) were performed according to the standard process. Images were acquired by a light microscope (Nikon, Japan). For adipocyte area analysis, 10 image fields per mouse were collected by Image-Pro Plus software (19). We manually selected for adipocytes (more than 250 cells per animal) and measured adipocyte area (20).

### Rectal Temperature Measurement and Cold Endurance Experiment

At the end of 4 weeks of EA treatment, the rectal temperature of the mice was recorded three times at 3 PM by an instrument at room temperature. Additionally, cold endurance experiment was performed as described previously (21). Mice were settled in a 4°C room, and rectal temperature was detected after 3, 6, 9, and 12 h.

### Intraperitoneal Glucose Tolerance Test

All mice were fasted for 12 h overnight at the end of EA treatment, and then glucose (2 g/kg BW) was administered intraperitoneally and blood glucose levels were measured at 0, 15, 30, 60, and 90 min.

### Metabolic Chamber Recordings

At the end of 4 weeks, 12 mice (3 each group) were given 2 days of acclimation in metabolic chambers before the trial and then continuously recorded for 24 h with the following measurements being taken every 40 min: food intake, water intake, ambulatory activity (in *X* and *Z* axes), and gas exchange (O_2_ and CO_2_). All measurements were taken automatically through the use of the LabMaster Phenotyping system (TSE PhenoMaster Systems, Germany). Oxygen consumption (VO_2_), carbon dioxide production (VCO_2_), heat production, and energy expenditure were calculated according to the manufacturer’s guidelines (PhenoMaster Software, TSE Systems). The respiratory exchange rate (RER) was estimated by calculating the ratio of VCO_2_/VO_2_.

### ELISA Detection of Serum Cholesterol, Triglyceride, Leptin, and LDL-c Level

Enzyme-linked immunosorbent assay (ELISA) kits were purchased from ShangHaiQiaDu Biotechnology Co. Ltd. Serum parameters were detected according to the manufacturer’s recommendations as described previously (18).

### Real-Time PCR Analysis

Total RNA was isolated from adipose tissue using Trizol reagent (Invitrogen, Cat#15596-026, USA) according to the manufacturer’s recommendations. RNA concentrations were quantified and synthesis of first-strand cDNA was reverse-transcribed using the ThermoScript^™^ RT-PCR System (Invitrogen, Cat#11146-016) (42°C, 1 h; 70°C, 5 min). The primer sequences are listed in **Table 1**. Real-time PCR (ViiA7 Real-time PCR, Life Technologies, USA) was performed with diluted cDNAs in a total reaction volume of 20 μl (per well) and measured in triplicate. Relative mRNA levels were calculated by ∆∆Ct and compared with housekeeper GAPDH as internal control. The cDNA was denatured at 95°C for 10 min followed by 40 cycles of PCR (95°C for 15 s, 60°C for 60 s).

**Table 1:** The sequences of experimental primers used for q-PCR.

Gene	Forward primer	Reverse primer
**Ucp1**	GGCCCTTGTAAACAACAAAATAC	GGCAACAAGAGCTGACAGTAAAT
**Pgc-1α**	ACCATGACTACTGTCAGTCACTC	GTCACAGGAGGCATCTTTGAAG
**Prdm16**	CCACCAGCGAGGACTTCAC	GGAGGACTCTCGTAGCTCGAA
**Teme26**	TGTTTGGTGGAGTCCTAAGGTC	ACCCTGTCATCCCACAGAG
**Tbx1**	GGCAGGCAGACGAATGTTC	TTGTCATCTACGGGCACAAAG
**β3ar**	ATCATGAGCCAGTGGTGGCGTGTAG	TCTAGTTCCCAGCGGAGTTTTATCG
**Gapdh**	GGCACAGTCAAGGCTGAGAATG	ATGGTGGTGAAGACGCCAGTA

### Western Blotting Analysis

Total proteins were extracted from the adipose tissue using the Total Protein Extraction Kit (Sigma, Cat# R0278). Protein concentrations were measured using the BCA Protein Assay Kit (Thermo scientific, Cat#23227). Twenty micrograms of proteins were resolved by 10% SDS-PAGE and transferred to PVDF membranes (Merck&millipore, Cat# SLGVV255F). Membranes were blocked with 5% bovine serum albumin (Merck&millipore, Cat#12659-500GM) in Tris-buffered saline with Tween 20 for 2 h followed by overnight incubation at 4°C with primary antibodies against UCP1 (Abcam, Cat#ab10983), PGC-1α (Santa Cruz, Cat#sc-13067), and PRDM16 (Abcam, Cat# ab106410). After three times washing with Tris buffered saline Tween20 (TBST), suitable HRP-labeled secondary antibody was incubated for 2 h at room temperature. Immunoblotting signals were visualized by ECL Kit (Thermo scientific). Bands were quantified by using the Image J software (NIH, Bethesda, MD, USA). Immunodetection of endogenous Glyceraldehyde-3-phosphate dehydrogenase (GAPDH) was utilized to estimate that equal amounts of protein were present in samples.

### Statistical Analysis

The data are presented as the mean ± standard deviation (SD) unless otherwise stated. All statistical analyses were performed using SPSS Version 17.0 statistic software. One-way repeated-measures analysis of variance (ANOVA) was used to compare the difference in BW among groups, followed by a *post hoc* Fisher test. Multiple group comparisons were made by one-way ANOVA, followed by the Turkey–Kramer HSD test. *P* < 0.05 was considered statistically significant between the comparing groups.

## Results

### EA Treatment Significantly Reduced Body Weight and Fat Accumulation

**Figure 1:**
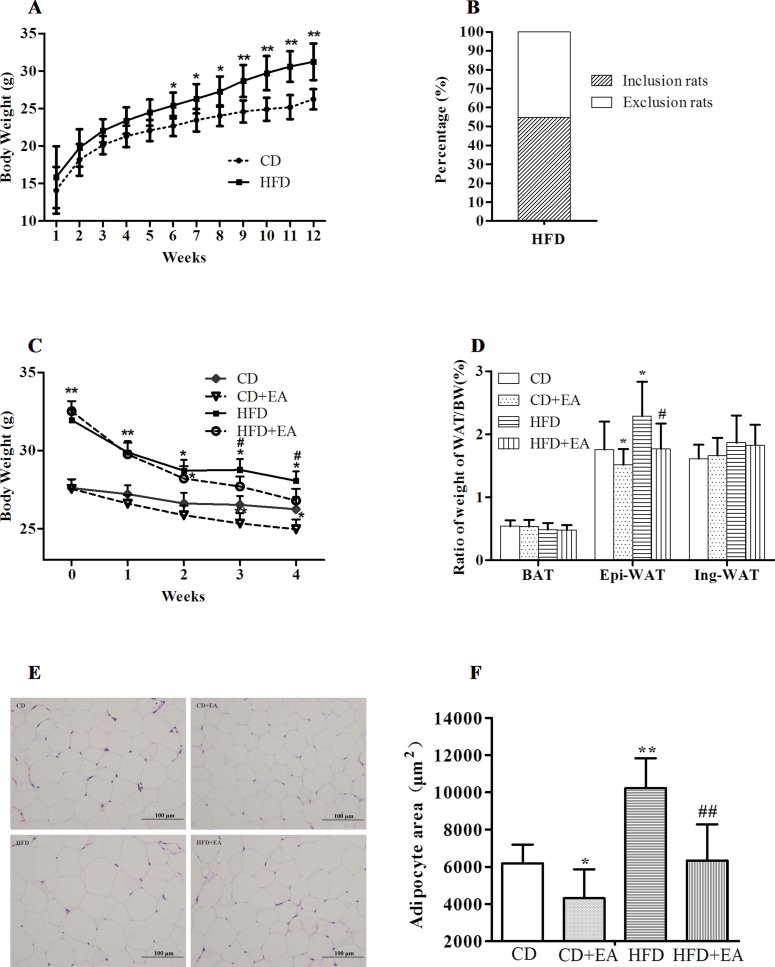
EA treatment significantly reduced body weight and fat accumulation. High-fat diet increased body weight **(A)**, and mice weigh more than 20% of the average BW of the CD group **(B)**. Mice were fed a common diet (CD) and high-fat diet (HFD) for 12 weeks. EA decreased body weight **(C)** and Epi-WAT weight **(D)**. Furthermore, EA treatment can also decrease obese mice adipocyte size **(E** and **F)**. Data represent the mean ± SD of 8–14 animals per group besides morphological analysis (*n* = 5). *P < 0.05, **P < 0.01 vs. the CD group; #*P* < 0.05, ##*P* < 0.01 vs. the HFD group.

After 12 weeks of high-fat diet, compared with the CD group (26.2 ± 1.3 g), the BW of the HFD group (31.3 ± 2.4 g) increased significantly (**Figure 1A**), in which 23 mice (54.76%) reached more than 20% of the average BW of the CD group (**Figure 1B**). During the 4 weeks of EA intervention, BW of the mice was recorded weekly. We observed that EA significantly reduced the HFD mice’s BW (from 32.5 ± 0.7 to 26.8 ± 0.7 g), especially at weeks 3 and 4 (**Figure 1C**). Interestingly, EA treatment also reduced the BW of the CD mice to some extent (**Figure 1C**). Additionally, we measured weight of the Epi-WAT, Ing-WAT, and BAT from each group and calculated the ratio of each type of adipose tissue to the BW. The results indicated that the Epi-WAT volume in the HFD group (0.67 ± 0.05 g) was larger than that in the CD group (0.38 ± 0.08 g), and EA significantly decreased the Epi-WAT/BW ratio (1.77 ± 0.4% vs. 2.29 ± 0.5%, *P* < 0.05) (**Figure 1D**). Moreover, H&E staining showed that the size of adipocyte in mice of the HFD group was larger than those in the CD mice (*P* < 0.05), and EA notably decreased adipocytes’ area in HFD + EA group (10,231.87 ± 1,602.23 vs. 6,337.77 ± 1,950.30 μm^2^, *P* < 0.01). It is interesting that EA also affected the CD mice (*P* < 0.05) (**Figure 1E and F**).

### EA Treatment Boosts Energy Metabolism in Obese Mice

**Figure 2:**
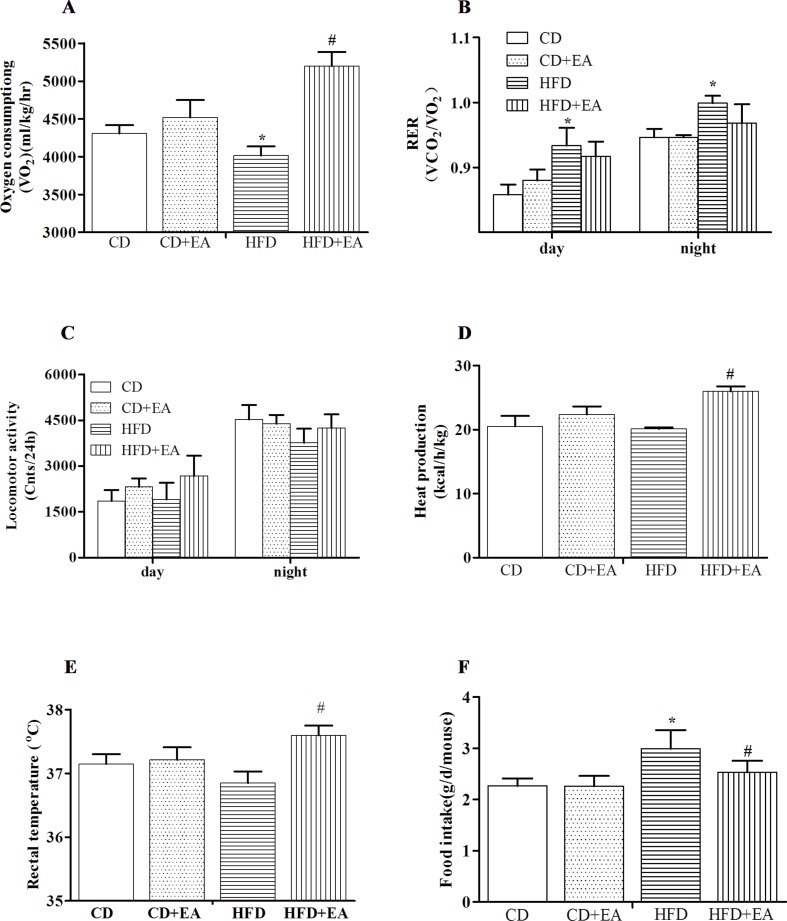
EA treatment affects metabolic phenotype in HFD mice. EA increased oxygen consumption **(A)**, but it did not affect the respiratory exchange rate **(B)** and locomotor activity **(C)**. Meanwhile, EA can increase heat production **(D)** and rectal temperature **(E)** and decrease food intake **(F)**. Data represent the mean ± SD of three animals per group. *P < 0.05 vs. the CD group; #*P* < 0.05 vs. the HFD group.

For investigating how EA treatment affected energy expenditure, indirect calorimetry, considered the gold standard for the assessment of resting energy expenditure, was performed by measuring oxygen consumption and carbon dioxide production. The results showed that EA significantly increased oxygen consumption (5,201.99 ± 182.06 vs. 4,015.09 ± 121.77, *P* < 0.05) over a 24-h period (**Figure 2A**), whereas there was no statistically significant difference in RER between the HFD group and the HFD + EA group (**Figure 2B**). Similarly, EA did not affect the locomotor activity (**Figure 2C**). Furthermore, heat production and rectal temperature (37.7 ± 0.2°C vs. 36.9 ± 0.2°C, *P* < 0.05) were markedly increased by EA treatment (**Figure 2D and E**), and EA also decreased the food intake (2.99 ± 0.36 g vs. 2.53 ± 0.23 g, *P* < 0.05) of obese mice (**Figure 2F**).

### EA Treatment Significantly Reversed Impaired Glucose Tolerance, Serum Leptin, Cholesterol, Triglyceride, and Insulin Level of Obese Mice

**Figure 3:**
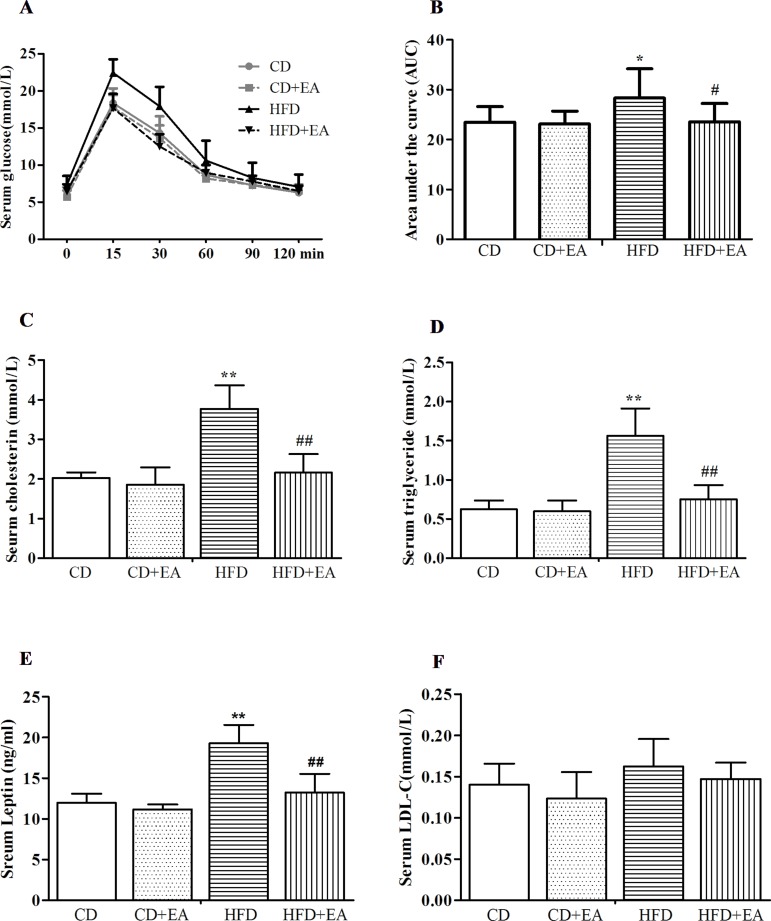
EA significantly reversed impaired glucose tolerance **(A)** and decreased the area under the curve (AUC) **(B)**. Meawhile, EA can decreased serum cholesterol **(C)**, TG **(D)** and leptin **(E)** levels, but it did not affect the serum LDL-c **(F)** level. Data represent the mean ± SD of five animals per group. *P < 0.05, **P < 0.01 vs. CD; #*P* < 0.05, ##*P* < 0.01 vs. HFD.

EA treatment reduced blood glucose levels during the Intraperitoneal glucose tolerance test (IGTT) in obese mice. As shown in **Figure 3A**, after 4 weeks of EA treatment, compared with other groups, the serum glucose in the HFD group increased significantly within 15 min (22.44 ± 1.83 mmol/L) after intraperitoneal glucose, while the decline was slower within 30 min (17.93 ± 2.6 mmol/L) and 60 min (10.62 ± 2.7 mmol/L). After further calculation of area under the curve (AUC), the results showed that the AUC of the serum blood glucose at 120 min was obviously increased in HFD mice in comparison with CD mice (**Figure 3B**). EA decreased the AUC in comparison with HFD mice (**Figure 3B**). Meanwhile, the serum levels of cholesterol, triglyceride (TG), leptin, and LDL-c were detected by ELISA kit, and the results indicated that, compared with CD mice, cholesterol (2.03 ± 0.14 vs. 3.77 ± 0.6 mmol/L), TG (0.62 ± 0.11 vs. 1.56 ± 0.35 mmol/L), and leptin (11.97 ± 1.13 vs. 19.29 ± 2.27 mmol/L) levels were all significantly elevated in HFD mice; however, 4 weeks of EA treatment completely reversed these levels to normal (3.77 ± 0.6 vs. 2.17 ± 0.47 mmol/L, 1.56 ± 0.35 vs. 0.75 ± 0.18 mmol/L, and 19.29 ± 2.27 vs. 13.22 ± 2.37 mmol/L, respectively), just as in mice of the CD group (**Figure 3C–E**). However, it did not change the serum LDL-c level in HFD mice.

### EA Treatment Induced the Expression of Thermogenesis-Associated Genes and Encoded Proteins in Adipose Tissue

**Figure 4:**
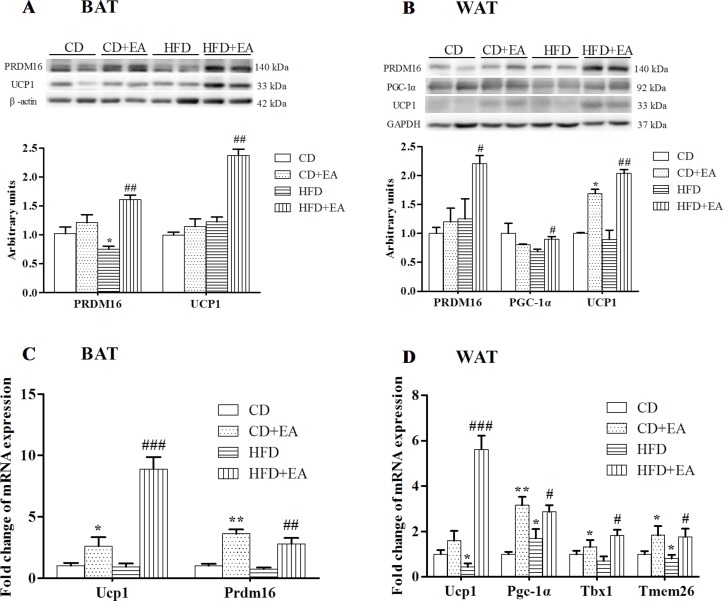
EA treatment promoted adipose tissue plasticity in HFD mice. EA treatment induced UCP1 and PRDM16 protein expression in BAT **(A)** and Epi-WAT **(B)**. EA also induced UCP1 and PRDM16 mRNA expression in BAT **(C)** and Epi-WAT **(D)**. EA induced beige adipocyte-specific gene expression in Epi-WAT **(D)**. Data represent the mean ± SD of five to six animals per group. *P < 0.05, **P < 0.01 vs. CD; #*P* < 0.05, ##*P* < 0.01, ###*P* < 0.001 vs. HFD.

The 4-week EA treatment promoted thermogenesis-associated genes and proteins in obese mice. The results showed significantly increased expression levels of UCP1 mRNA and protein in BAT after EA treatment (**Figure 4A and C**). Moreover, UCP1 mRNA and protein expression was also enhanced in the Epi-WAT in the HFD + EA group (**Figure 4B and D**). In addition, the expression levels of beige adipocyte marker genes, including peroxisome proliferator-activated receptor γ coactivator 1α (Pgc1α), PR domain containing 16 (Prdm16), and Tmem26, were elevated in BAT and WAT of mice treated by EA (**Figure 4C and D**). Interestingly, T-box transcription factor 1 (Tbx1), recently defined as the beige adipocyte marker, was also significantly induced in WAT by EA treatment (**Figure 4D**).

### EA Treatment Activated Sympathetic Nerves of WAT in HFD Mice

**Figure 5:**
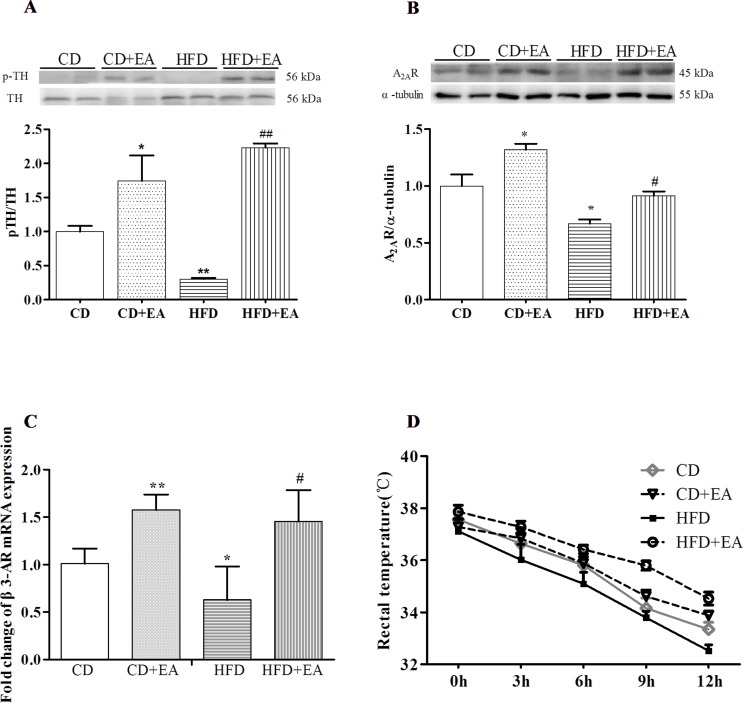
EA treatment activated sympathetic nerves of WAT in HFD mice. EA treatment promoted p-TH **(A)** and A2AR **(B)** protein expression in Epi-WAT. EA also increased the β3-AR mRNA expression in Epi-WAT **(C)**. Moreover, EA can affect the ability of cold endurance **(D)**. Data represent the mean ± SD of five to six animals per group. *P < 0.05, **P < 0.01 vs. CD; #*P* < 0.05, ##*P* < 0.01 vs. HFD.

After 4 weeks of EA treatment, sympathetic activation-related protein expression increased in WAT of obese mice. Compared with the CD group, the expression of p-TH and A_2A_R decreased in the HFD group, but were reversed by EA treatment (**Figure 5A and B**). Moreover, EA also promoted the expression of β3 AR mRNA level of Epi-WAT (**Figure 5C**). Meanwhile, we also observed that EA increased rectal temperature of obese mice at 12 h markedly but did not affect that of CD mice (**Figure 5D**), indicating that EA treatment could affect the autonomic nervous system of obese mice and enable them to adapt to cold temperature.

## Discussion

Mammals have three types of adipose tissues: white, brown, and beige adipose. WAT is the main tissue of energy storage, while BAT is specialized for dissipating chemical energy by generating heat to maintain adequate core body temperature. Beige adipose is genetically different from both BAT and WAT but burns calories to release energy like BAT. Some factors such as cold exposure, SNS activation, and pharmacological conditions recruit a distinct type of thermogenic fat cell called beige adipocytes to the white fat through a process called “browning” (12, 22). Generally, WAT is characterized by its metabolic and endocrine functions for regulating energy homeostasis and insulin sensitivity. However, in the context of sustained obesity, WAT undergoes fibro-inflammation, which compromises its functionality, contributing to increased risk of type 2 diabetes and chronic cardiovascular conditions. Conversely, improving adipose tissue plasticity, either by expanding anabolic functions of WAT or by increasing tissue thermogenesis through activation of pre-existing BAT, and inducing beige adipocyte formation represent potential therapeutic approaches (23, 24). In this study, our results show that EA stimulation can significantly restore obese phenotype, promote adipose tissue plasticity, and activate sympathetic excitability in obese mice. Additionally, EA can also induce adipose tissue plasticity *via* promoting the expression of fat browning-related proteins, such as UCP-1, PRDM16, and PGC-1α in adipose tissue.

Consistent with findings from previous studies, EA treatment prevented the development of obesity (21, 25–28); however, the mechanisms remain unclear. The brain has always been a hot spot in acupuncture weight loss research; however, as the main target organ of obesity, adipose tissue has not gained enough attention. Growing body of evidence shows that inducing the formation of beige fat or WAT browning can reduce diet-induced BW gain and control obesity-related diseases (15, 29, 30). The most important characteristic of WAT browning is the elevated expression of UCP1, which is specifically BAT marker genes. As a major determinant to BAT thermogenic activity, any increase in UCP1 is commonly considered as the trademark of energy expenditure (31). Similar to UCP1, PGC-1α is also involved in the process of browning WAT (32). As a transcriptional coactivator of the nuclear receptor PPARγ, it is considered to be an integral regulator of genes that participate in mitochondrial biogenesis and oxidative metabolism (33). Moreover, increased expression levels of PRDM16 in white adipocyte precursors induce a full brown adipocyte gene programming and stimulate both mitochondrial biogenesis and uncoupled cellular respiration (34), and ablation of PRDM16 caused metabolic dysfunction (35). Browning of WAT or the recruitment of beige adipocytes can be brought about by hormones, cytokines, nutrients, and drugs (31, 33, 36). Our results show that 4 weeks of treatment with EA can promote WAT and BAT plasticity in obese mice through inducing the expression of UCP1 and PRDM16, thus promoting WAT lipolysis and decreasing adipocyte size. Meanwhile, PGC-1α, TMEM26, and TBX1, as beige markers (37), are greatly induced in WAT by EA. Since UCP1 and PRDM16 are responsible for energy dissipation *via* nonshivering thermogenesis, mice with higher UCP1 and PRDM16 expression can generate heat more efficiently to maintain body temperature under cold circumstances. Therefore, impaired glucose tolerance improved in obese mice. Meanwhile, with the WAT browning, serum leptin level is significantly decreased, as did the TG and cholesterol levels. Our experiment provides evidence of adipose tissue plasticity by EA on obese mice.

Historically, the control of WAT lipolysis has mainly focused on the adrenal medullary catecholamines epinephrine (EPI) and norepinephrine (NE). Existing research indicates that WAT is innervated by SNS, and its activation is responsible for lipolysis in WAT (38, 39); nevertheless, parasympathetic innervation is not supported (38). Fully executed SNS-NE-mediated WAT lipolysis is dependent on β-adrenoceptors (βARs), especially the β3AR, which received significant attention (40, 41). This subtype is predominantly expressed on white and brown adipocytes in rodents and on brown adipocytes in humans, and its selective ligands have marked anti-obesity actions in rats and mice (42). Moreover, the SNS is fundamental in the control of daily energy expenditure *via* the regulation of resting metabolic rate and thermogenesis in response to physiologically relevant stimuli, that is, changing energy states, carbohydrate consumption, food intake, hyperinsulinemia, and cold exposure (43). Tyrosine hydroxylase (TH), a marker of sympathetic nerves, reflects the density of nerve fibers effectively (39, 44). Additionally, the adenosine 2A receptor (A2AR), as the most abundant adenosine receptor in adipose tissue, was affected during stimulation of sympathetic nerves. Pharmacological stimulation of A2AR or injection of lentiviral vectors overexpressing the A2AR into white fat induces brown-like cells, which are called beige adipocytes (15). Importantly, mice fed an HFD and treated with an A2AR agonist are leaner, with improved glucose tolerance and increased energy expenditure (15). Our results support that EA can increase the expression of TH, A2A receptor, and β3AR mRNA in WAT. At the same time, it also enhances the body’s ability to tolerate cold exposure. The findings suggest that the activity of SNS in obese mice could be increased after 4 weeks of EA treatment.

Of course, this study also has some limitations. First, the number of examined cases is modest, which will limit the possibility of drawing generalizable conclusions to some extent. Second, EA, which is characterized by partial electrical stimulation, could induce muscle contraction and consequently consume energy, which is similar to physical exercise to a certain extent. Emerging evidence showed that muscle contraction causes an immediate increased glucose uptake in skeletal muscle and adipose tissue (45, 46), although EA can increase the whole-body glucose uptake by activated autonomic nervous system (47). We cannot rule out the anti-obesity effects of muscle contractions induced by EA in this study. In addition, HFD can induce the inhibition of sympathetic outflow to BAT (48), and future studies will observe sympathetic activity in BAT and evaluate WAT sympathetic drive measured by electrophysiological and neurochemical (NE turnover) means (38). In particular, the exact mode of signaling by which leptin triggers changes in WAT function was yet to be identified, and it also showed that the SNS is the fine effector of leptin’s action on WAT (39). Serum leptin level increased after 4 weeks of EA treatment in this study, suggesting that leptin may be involved in the SNS-mediated adipose tissue plasticity, but it requires further experimentation to reveal the hypothesis.

## Conclusions

Taken together, EA treatment enhances sympathetic nerve activity *via* activation of TH and A2AR, thereby promoting adipose tissue plasticity and increasing energy expenditure through inducing UCP1 and PRDM16 expression, and this may be one of the mechanisms by which EA treatment decreases BW gain and fat accumulation.

## Data Availability Statement

All datasets generated for this study are included in the manuscript and the supplementary files.

## Ethics Statement

The study was approved by the Institutional Animal Care and Use Committee of Nanjing University of Chinese Medicine, and all procedures were conducted in accordance with the guidelines of the NIH Animal Care and Use Committee.

## Author Contributions

B-MZ, S-FL, and M-LY conceived and designed the experiments. S-FL, Y-XT, TZ, S-PF, HH, YC, and X-YJ performed the experiments. Y-XT, S-FL, M-LY, and H-XX analyzed the data. S-FL, M-LY, and B-MZ wrote the paper. All authors have read and agreed with the manuscript.

## Funding

This work was supported by the National Natural Science Foundation of China (Nos. 81273838, 81303019 and 81574062), Jiangsu Province “333 High-level Talents Cultivating Project” (2016), “Six Major Talent Summit” of Jiangsu Province (YY-033), “Qing Lan Project” of Jiangsu Province (2016), and Key University Science Research Project of Jiangsu Province (Nos. 16KJA360003 and 17KJA360001).

## Conflict of Interest Statement

The authors declare that the research was conducted in the absence of any commercial or financial relationships that could be construed as a potential conflict of interest.
